# Systemische immunmodulierende Therapien der epidermalen Nekrolyse (Stevens‐Johnson‐Syndrom/toxisch epidermale Nekrolyse): Systematische Übersicht und Metaanalyse

**DOI:** 10.1111/ddg.15804_g

**Published:** 2026-01-14

**Authors:** Ruben Heuer, Maren Paulmann, Maja Mockenhaupt, Alexander Nast

**Affiliations:** ^1^ Division of Evidence‐based Medicine (dEBM) Klinik für Dermatologie Venerologie und Allergologie Charité – Universitätsmedizin Berlin Corporate Member of Free University of Berlin Humboldt University of Berlin and Berlin Institute of Health, Berlin; ^2^ Dokumentationszentrum schwerer Hautreaktionen (dZh) Klinik für Dermatologie und Venerologie Universitätsklinikum Freiburg

**Keywords:** Arzneimittelreaktion, Stevens‐Johnson‐Syndrom, Systemtherapie, Systematischer Review, Toxisch epidermale Nekrolyse, drug reaction, epidermal necrolysis, stevens‐Johnson syndrome, systematic Review, toxic epidermal necrolysis

## Abstract

**Hintergrund und Ziele:**

Epidermale Nekrolyse ist eine seltene, aber schwere Hautreaktion mit hoher Letalität. Zur Wirksamkeit systemischer immunmodulatorischer Therapien (SIT) liegt wenig Evidenz vor. Ziel unserer systematischen Übersichtsarbeit war der Vergleich von SIT mit supportiver Therapie oder untereinander.

**Patienten und Methoden:**

Es wurden randomisierte kontrollierte Studien und kontrollierte Beobachtungsstudien (≥ 5 Patienten jeden Alters pro Behandlungsarm) unter Verwendung der internationalen Konsensus‐Klassifikation für EN (Bastuji‐Garin, 1993) ohne signifikante Unterschiede der Ausgangswerte eingeschlossen. Wir durchsuchten MEDLINE, Embase und Cochrane CENTRAL (1. Januar 1993 bis 22. Januar 2024). Zwei Untersucher extrahierten unabhängig voneinander Studiencharakteristika und Ergebnisdaten. Eine *Random‐Effects*‐Metaanalyse wurde durchgeführt.

**Ergebnisse:**

Der primäre Endpunkt war die Letalität. Sekundäre Endpunkte umfassten Krankenhausaufenthaltsdauer, Zeit bis zur vollständigen Reepithelisierung, Komplikationen und Folgeerscheinungen. 43 Studien mit 58 Behandlungsvergleichen wurden ausgewertet. Im Vergleich zur supportiven Therapie zeigte lediglich Etanercept einen signifikanten Letalitätsvorteil (RR 0,32, 95%‐KI 0,11–0,93; GRADE: unzureichende Präzision/unklare klinische Bedeutung). Ciclosporin war IVIG hinsichtlich der Letalität überlegen (RR 0,18, 95%‐KI 0,05–0,58), und Kortikosteroide plus IVIG reduzierten schwere Komplikationen im Vergleich zu Kortikosteroiden allein (Sepsis, RR 0,77, 95%‐KI 0,31–0,77). Im Vergleich zur supportiven Therapie zeigte lediglich Etanercept einen signifikanten Letalitätsvorteil (RR 0,32, 95%‐KI 0,11–0,93; GRADE: unzureichende Präzision/unklare klinische Bedeutung).

**Schlussfolgerungen:**

Es gibt keine eindeutigen Belege für die Überlegenheit von SIT gegenüber der supportiven Behandlung, die nach wie vor die primäre Behandlung bei EN darstellt.

## EINLEITUNG

Die epidermale Nekrolyse (EN; auch Stevens‐Johnson‐Syndrom/toxisch epidermale Nekrolyse) ist eine akute, lebensbedrohliche mukokutane Reaktion mit hoher Letalität.^1^, ^2^ Charakteristisch für die epidermolytischen Nekrolysen (EN) sind Epidermisablösungen und Schleimhauterosionen, deren Schweregrad anhand des Ausmaßes der Epidermisablösung klassifiziert wird: Stevens‐Johnson‐Syndrom (SJS; <10%), toxisch epidermale Nekrolyse (TEN; >30%) sowie eine Übergangsform zwischen SJS und TEN mit einem Ablösungsgrad von 10–30%.^3^ Die epidermale Nekrolyse ist selten, die Gesamtinzidenz beträgt in Deutschland 0,93 Fälle pro 1 Million Personen pro Jahr (95%‐Konfidenzintervall [KI]: 0,86–1,00).^2^ Aufgrund der sehr niedrigen Inzidenz und der erheblichen Heterogenität der Patientenpopulation sind Durchführung und Interpretation von Studien zur EN eine Herausforderung.

Die höchste Priorität in der klinischen Versorgung besteht darin, supportive Maßnahmen einzuleiten und die wahrscheinlichste Ursache zu identifizieren. Da EN hauptsächlich medikamenteninduziert auftritt,^4^, ^5^, ^6^ ist das Absetzen des potenziell auslösenden Medikaments unerlässlich.^7^ Zusätzlich zur supportiven Behandlung werden verschiedene systemische immunmodulierende Therapien (SIT) eingesetzt. Kortikosteroide, intravenöse Immunglobuline (IVIG), Ciclosporin, Tumornekrosefaktor (TNF)‐α‐Inhibitoren (zum Beispiel Etanercept, Infliximab) oder eine Kombination verschiedener SIT werden in der klinischen Praxis routinemäßig verabreicht.^8^, ^9^, ^10^, ^11^

Frühere Metaanalysen zeigten keinen signifikanten Überlebensvorteil für Kortikosteroide,^8^, ^9^, ^12^ IVIG,^8^, ^9^, ^12^, ^13^ Ciclosporin,^9^, ^12^ oder die Kombination von Steroiden mit IVIG,^12^ im Vergleich zur supportiven Therapie. Zusätzlich zum Vergleich einer Intervention mit supportiver Therapie verwenden einige Metaanalysen den SCORTEN (*severity‐of‐illness score for TEN*),^14^ um eine standardisierte Letalitätsrate (SMR; Quotient aus beobachteter und durch den SCORTEN vorhergesagter Letalität) zu berechnen.^15^, ^16^, ^17^, ^18^ Direkte Vergleiche sind jedoch deutlich seltener.

Diese systematische Übersichtsarbeit und Metaanalyse wurde im Rahmen der Entwicklung der deutschen evidenzbasierten Leitlinie zur Diagnostik und Therapie der EN durchgeführt. Nach Fertigstellung der Leitlinie erfolgte eine weitere aktualisierte Suche. Ziel der Übersicht ist es, *(1)* einen umfassenden Überblick über vorgeschlagene SIT zu geben und *(2)* deren Effekte auf verschiedene Endpunkte im Vergleich zur supportiven Therapie oder zu den anderen eingeschlossenen SIT abzuschätzen.

## PATIENTEN UND METHODIK

Wir folgten den von Higgins et al. empfohlenen Methoden systematischer Übersichtsarbeiten.^19^ Für jeden Vergleich wurde die Sicherheit der berichteten Effektschätzung gemäß den Richtlinien der Arbeitsgruppe *Grading of Recommendations Assessment, Development and Evaluation* (GRADE) bewertet (methodische Details in der Tabelle S1 im Online‐Supplement).^20^ Wir berichteten unsere Ergebnisse gemäß der PRISMA‐Richtlinie (*Preferred Reporting Items for Systematic Reviews and Meta‐Analyses*). Vor der Datenerhebung und ‐analyse wurde das Protokoll auf PROSPERO registriert (Registrierungsnummer: CRD42023423396).

### Suchstrategie und Auswahl‐/Eignungskriterien

Am 3. Mai 2023 durchsuchten wir MEDLINE und Embase (Suchbegriffe in Tabelle S2 im Online‐Supplement) nach Primärstudien zu Interventionen. Eine erneute Suche erfolgte am 22. Januar 2024 (Embase, MEDLINE) und am 5. April 2024 (Cochrane CENTRAL).

Um das Risiko zu verringern, Studien mit Nicht‐EN‐Patienten einzuschließen, beschränkten wir den Suchzeitraum auf den Veröffentlichungsmonat der etablierten Krankheitsklassifikation von Bastuji‐Garin et al. ab Januar 1993, da bis dahin eine eindeutige Krankheitsdefinition fehlte.^3^ Aus demselben Grund mussten die eingeschlossenen Studien entweder auf diese Publikation verweisen oder eine vergleichbare Krankheitsklassifikation angeben (zum Beispiel RegiSCAR‐Kriterien).

Wir schlossen alle randomisierten, kontrollierten Parallelgruppenstudien und nichtrandomisierten, kontrollierten Parallelgruppenstudien mit mindestens fünf Teilnehmern aller Altersgruppen pro Behandlungsarm ein. Ebenfalls schlossen wir unkontrollierte Kohortenstudien mit mindestens zwei systemischen immunmodulatorischen Therapien und mindestens fünf Teilnehmern pro Behandlungsarm ein.

Studien, deren Informationen unzureichend waren, um ein zusammenfassendes Effektmaß für mindestens ein relevantes Ergebnis zu extrahieren oder zu berechnen, wurden ausgeschlossen. Das galt auch für Studien mit unvollständigen Basischarakteristika, bei denen die berichteten Effektschätzer nicht adjustiert waren (zum Beispiel über multiple Regressionsanalyse). Als unvollständige Berichterstattung betrachteten wir fehlende Informationen zum Schweregrad der Erkrankung (zumindest die betroffene Gesamtkörperoberfläche oder SCORTEN) und zur Komorbidität (zumindest das Alter der Patienten als Näherungsmaß). Wir verglichen die Basischarakteristika zwischen allen Studien und schlossen Studien mit klinisch bedeutsamen Unterschieden aus, operationalisiert als Unterschiede im Durchschnittsalter > 15 Jahre, der durchschnittlichen betroffenen Körperoberfläche >15% und der durchschnittlichen erwarteten Letalität (nach SCORTEN) >30%. Für den Ausschluss war zudem statistische Signifikanz (p <0,05) der Unterschiede zwischen den Gruppen erforderlich, die durch geeignete Hypothesentests (parametrische Tests für aggregierte Effektschätzer, nichtparametrische Tests für Studien, die auf Patientenebene berichten) ermittelt wurde.

Es wurde keine Sprachbeschränkung angewendet. Referenzen in den enthaltenen Artikeln und Publikationen auf lens.org, die auf die enthaltenen Artikel verweisen, wurden überprüft, um potenziell übersehene Studien zu identifizieren (Vorwärts‐ und Rückwärtssuche). Zur Verwaltung der Referenzen wurde Endnote verwendet.

### Studienauswahl und Datenextraktion

Zwei Gutachter (MP und RH) führten unabhängig voneinander die Studienauswahl und Datenextraktion durch; Meinungsverschiedenheiten wurden durch Diskussion geklärt. Die Studienauswahl erfolgte in zwei Schritten: *(1)* Sichtung von Titel und Abstract und *(2)* Auswertung des Volltextes. Bei fehlenden Basischarakteristika und vergleichenden Ergebnissen wurden, sofern verfügbar, individuelle Patientendaten zur Berechnung dieser Kennzahlen herangezogen.

### Datensynthese/‐analyse

Soweit möglich, fassten wir die Daten aus einzelnen Studien quantitativ zusammen und führten eine Metaanalyse durch, wenn mindestens zwei Studien über denselben Endpunkt für dieselbe Gruppe von Interventionen berichteten. Für dichotome Ergebnisse (Letalität, unerwünschte Ereignisse) wurden *Risk Ratios* (RR) geschätzt. Für kontinuierliche Endpunkte berechneten wir nicht standardisierte, gepoolte Mittelwertdifferenzen, da alle eingeschlossenen Studien Schätzwerte auf derselben Skala berichteten (Zeit bis zur vollständigen Reepithelisierung und Dauer des Krankenhausaufenthalts in Tagen). Wo lediglich Standardfehler (SE) oder Konfidenzintervalle für kontinuierliche Ergebnisse verfügbar waren, berechneten wir die Standardabweichungen mithilfe etablierter Verfahren.^19^ Wir verwendeten R Version 4.3.1 (*The R Foundation for Statistical Computing Platform*), um Mittelwertdifferenzen (MD) und RR mit den entsprechenden 95%‐Konfidenzintervallen (KI) zu berechnen. Die dargestellten zusammenfassenden Maße basieren auf einem *Random‐Effects*‐Modell (DerSimonian‐Laird), da aufgrund der methodischen Heterogenität und historisch unterschiedlicher Behandlungspraktiken (beispielsweise supportive Behandlungstraditionen) zwischen Ländern und Behandlungszentren studienspezifische wahre Effekte angenommen wurden. Da auch ein *Fixed‐Effects*‐Modell unter Verwendung der Mantel‐Haenszel‐Methode in Betracht gezogen werden könnte, stellten wir dessen Ergebnisse in *Forest‐Plots* und Tabellen dar, um mögliche Abweichungen zwischen den beiden Ansätzen aufzuzeigen.

Um die Robustheit der gepoolten Schätzungen zu überprüfen, führten wir die folgenden Sensitivitätsanalysen durch: *(1)* Variation des Schwellenwerts für die klinische Signifikanz von Unterschieden zwischen den Gruppen zu Studienbeginn (α‐Wert), *(2)* Ausschluss von Studien mit hohem Verzerrungspotenzial, *(3)* Ausschluss von Studien ohne Todesfälle in einem der Interventionsarme, *(3)* Variation der Methode zur Berechnung des τ^2^‐Schätzers.

## ERGEBNISSE

### Literaturrecherche und Charakteristika der eingeschlossenen Studien

Die Datenbanksuche ergab 10 829 Referenzen (Duplikate ausgeschlossen). Nach Ausschluss thematisch unzutreffender oder nicht auffindbarer Publikationen wurden 1091 Artikel im Volltext ausgewertet. Schließlich wurden 43 Studien, die die Auswahlkriterien erfüllten, in die Übersichtsarbeit aufgenommen (PRISMA‐Flussdiagramm siehe Abbildung S1 im Online‐Supplement). Charakteristika aller eingeschlossenen Studien sind in der Tabelle S3 im Online‐Supplement dargestellt. Insgesamt lieferten Studien aus 17 Ländern – darunter zwei randomisierte kontrollierte Therapiestudien (RCT) sowie 42 beobachtende Kohortenstudien (4 prospektive, 38 retrospektive, einschließlich eines ergänzenden Vergleichs aus einer der RCT auf Basis historischer Daten) – insgesamt 21 direkte Paarvergleiche. Nur zwei Studien berichteten ausschließlich über die Behandlung von Kindern,^21^, ^22^ während sich die übrigen auf Erwachsene konzentrierten oder Patienten aller Altersgruppen einschlossen. Alle Studien beschreiben mindestens zwei unterschiedliche Behandlungsarme und die Patienten wurden entweder ausschließlich supportiv (21), mit Kortikosteroiden (26), IVIG (14), Ciclosporin (8), anderen SIT als Monotherapie (4) oder mit einer Kombination aus zwei SIT (19) behandelt.

**ABBILDUNG 1:**
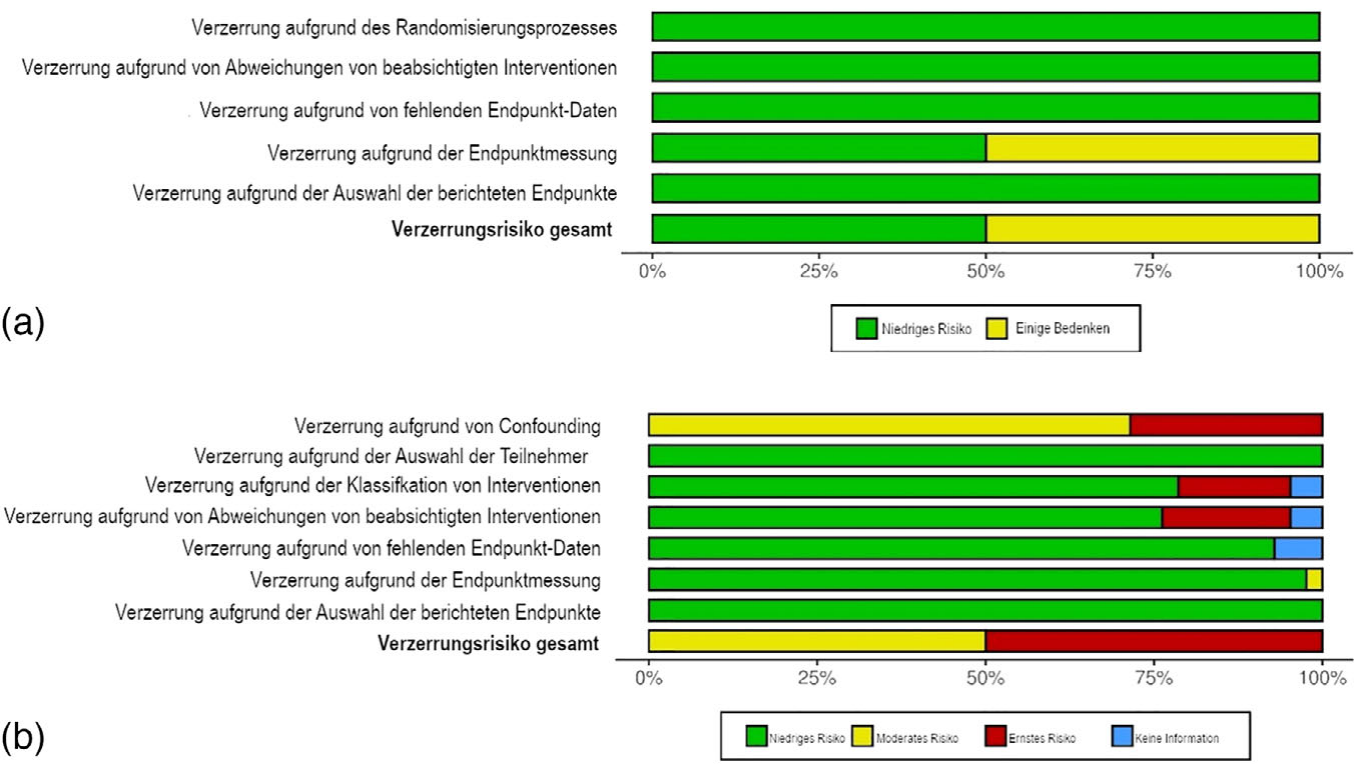
Zusammenfassung des Verzerrungsrisikos. Die Diagramme wurden mit Robvis erstellt.⁷⁹ (a) Bewertung randomisierter kontrollierter Studien mit dem Cochrane Risk of Bias Tool 2.0 (b) Bewertung nichtrandomisierter Studien mit ROBINS‐I.

Das Verzerrungsrisiko wurde für alle Endpunkte bewertet und separat für RCT *(a)* in Abbildung 1 (detaillierte Auswertung in Abbildung S2 im Online‐Supplement) und für nichtrandomisierte Studien *(b)* in Abbildung 1 (detaillierte Auswertung in Abbildung S3 im Online‐Supplement) dargestellt. Wir verwendeten das RoB 2‐Tool zur Bewertung der beiden RCT, von denen eine mit geringem Verzerrungsrisiko,^23^ und eine mit einigen Bedenken hinsichtlich der Ergebnismessung,^24^ bewertet wurde. Mit dem ROBINS‐I‐Instrument beurteilten wir 42 Beobachtungsstudien, von denen die Hälfte eine Gesamtbewertung von moderat oder hoch erhielt.^21^, ^22^, ^24^, ^25^, ^26^, ^27^, ^28^, ^29^, ^30^, ^31^, ^32^, ^33^, ^34^, ^35^, ^36^, ^37^, ^38^, ^39^, ^40^, ^41^, ^42^, ^43^, ^44^, ^45^, ^46^, ^47^, ^48^, ^49^, ^50^, ^51^, ^52^, ^53^, ^54^, ^55^, ^56^, ^57^, ^58^, ^59^, ^60^, ^61^, ^62^, ^63^

Ursprünglich wurde geplant, die Studien nach dem Alter der Patienten zu stratifizieren, die Daten reichten jedoch für eine Untergruppenanalyse nicht aus.

### Letalität

**ABBILDUNG 2:**
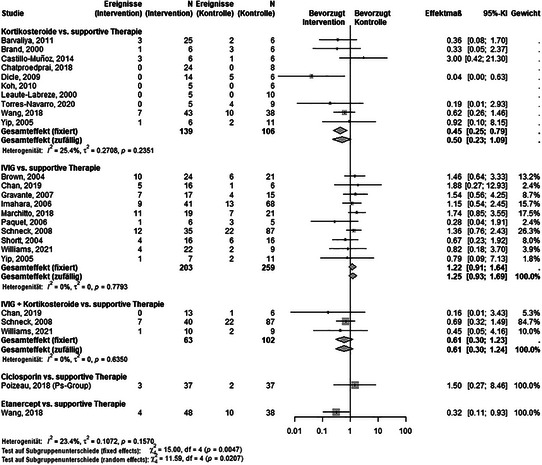
Vergleich verschiedener systemischer immunmodulierender Therapien mit supportiver Therapie im Hinblick auf die Letalität.

Es gab sechs direkte paarweise Vergleiche zum Effekt von SIT gegenüber supportiver Therapie bezogen auf die Letalität (Abbildung S2 und Tabelle S4 im Online‐Supplement). Abbildung 2 zeigt die am häufigsten eingesetzten SIT, weitere Mono‐ oder Kombinationstherapien finden sich im Supplement. Mittels eines *Random‐Effects*‐Modells konnte für Kortikosteroide kein statistisch signifikanter Behandlungseffekt nachgewiesen werden (RR 0,5; 95%‐KI 0,23–1,09). Ein Vergleich von Etanercept mit supportiver Therapie ergab einen statistisch signifikanten Vorteil für die Intervention (RR 0,32; 95%‐KI 0,11–0,93; GRADE: unzureichende Präzision aufgrund weiter Konfidenzintervalle/unklarer klinischer Relevanz). Die übrigen SIT zeigten keinen statistisch signifikanten Effekt im Vergleich zur supportiven Therapie.

**ABBILDUNG 3:**
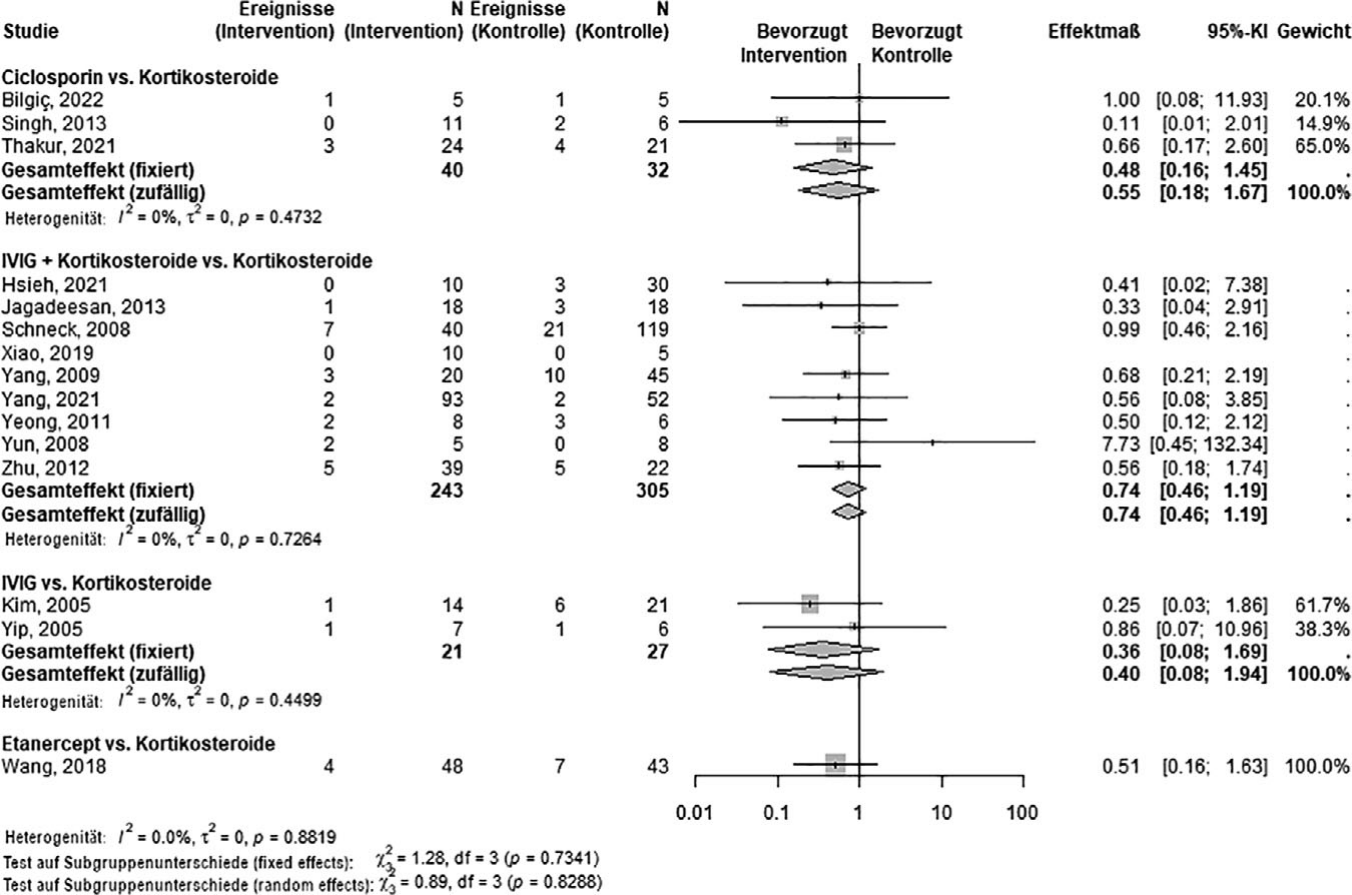
Vergleich verschiedener systemischer immunmodulierender Therapien mit Kortikosteroiden hinsichtlich der Letalität.

Es gab sieben direkte Paarvergleiche von SIT mit Kortikosteroiden (Abbildung 3 und Tabelle S4 im Online‐Supplement). Innerhalb dieser Vergleichsgruppe zeigte keine Intervention einen statistisch signifikanten Unterschied (Abbildung 3).

Im Gegensatz dazu wurde in folgenden direkten Vergleichen ein statistisch signifikanter Effekt beobachtet: Ciclosporin versus IVIG (RR 0,18, 95%‐KI 0,05–0,58), Kortikosteroide plus IVIG versus IVIG (RR 0,46, 95%‐KI 0,22–0,96) und Thalidomid zugunsten von Placebo (RR 2,78, 95%‐KI 1,04–7,4) (Tabelle S4 im Online‐Supplement).

Bei allen Vergleichen erfüllte die Anzahl der eingeschlossenen Patienten pro Behandlungsarm nicht das *Optimal‐Information‐Size* (OIS)‐Kriterium. Die berichteten Effektschätzer müssen daher als unpräzise angesehen werden.

Visuelle Betrachtung der *funnel plots* und des Eggers‐Regressionstests für den einzigen möglichen Endpunkt Letalität ergaben keine statistischen Hinweise auf einen *small study bias*. Aufgrund der geringen Studienanzahl pro Metaanalyse konnten jedoch nur zwei Vergleiche ausgewertet werden. Ein *small study bias* kann daher nicht ausgeschlossen werden (Abbildung S4 im Online‐Supplement).

### Zeit bis zur vollständigen Reepithelisierung

Insgesamt wurden neun Studien für die Auswertung von sieben direkten Paarvergleichen hinsichtlich der Zeit bis zur vollständigen Reepithelisierung eingeschlossen (Tabelle S5 und Abbildung S5 im Online‐Supplement). Die Analyse von Ciclosporin im Vergleich zu Kortikosteroiden erfüllte das OIS‐Kriterium, offenbarte jedoch eine erhebliche Heterogenität (*I^2^* > 70%). Alle drei für diesen Vergleich identifizierten Studien zeigten einen Effekt zugunsten von Ciclosporin, wobei zwei Studien einen signifikanten Effekt^50^, ^52^ und eine Studie keine statistische Signifikanz aufwiesen.^53^ Im Vergleich zu Kortikosteroiden plus Cyclophosphamid führte die Anwendung von Ciclosporin zu einer klinisch signifikant kürzeren Zeit bis zur vollständigen Reepithelisierung (MD –5,7, 95%‐KI –9–(–2,4)), ohne das OIS‐Kriterium zu erfüllen.

Im Vergleich zu Kortikosteroiden allein verkürzte sich die Zeit bis zur vollständigen Reepithelisierung bei der Kombination aus Kortikosteroiden plus IVIG (MD –2,93, 95%‐KI –4,4–(–1,46; GRADE: unzureichende Präzision aufgrund großer Konfidenzintervalle/unklarer klinischer Relevanz)) und bei der Kombination aus Kortikosteroiden plus Adalimumab (MD –3,5, 95%‐KI –5,17–(–1,83); GRADE: unzureichende Präzision aufgrund großer Konfidenzintervalle/unklarer klinischer Relevanz). Bei allen anderen statistisch signifikanten Vergleichen überschritten die Konfidenzintervalle die klinische Entscheidungsschwelle, was ihre klinische Relevanz in Frage stellt.

### Dauer des Krankenhausaufenthaltes

Für die Auswertung von neun direkten Paarvergleichen zur Krankenhausverweildauer wurden insgesamt 21 Studien berücksichtigt (Tabelle S6 und Abbildung S6 im Online‐Supplement). Bei fünf direkten Paarvergleichen zeigte nur ein Vergleich einen statistisch signifikanten Unterschied (Kortikosteroide plus IVIG versus Kortikosteroide), der auf Studien mit erheblicher Heterogenität (*I^2^* >70%) beruhte. Die vier für diesen Vergleich identifizierten Studien wiesen einen Effekt zugunsten der Kombinationstherapie auf, wobei zwei Studien einen signifikanten Effekt zeigten,^56^, ^57^ zwei hingegen nicht.^40^, ^55^ Weitere Vergleiche mit IVIG, entweder allein oder in Kombination mit N‐Acetylcystein sowie die Kombination von Kortikosteroid plus Ciclosporin zeigten keinen statistisch signifikanten Unterschied.

Bei jedem Vergleich erfüllte die Anzahl der eingeschlossenen Patienten pro Behandlungsarm nicht das OIS‐Kriterium, sodass die berichteten Effektschätzungen ungenau sind.

### Schwerwiegende Komplikationen und Langzeitfolgen

Für die schwerwiegenden Komplikationen Sepsis, Organversagen und mechanische Beatmung wurden verschiedene Studien identifiziert und ausgewertet (Tabelle S7 im Online‐Supplement). Insgesamt wurden 15 Studien für acht direkte Paarvergleiche zu Sepsis, neun Studien für fünf Vergleiche zu Organversagen und sechs Studien für sechs Vergleiche zur mechanischen Beatmung eingeschlossen. Nur Kortikosteroide zeigten im Vergleich zur supportiven Therapie einen statistisch signifikanten Effekt (schwerwiegende Komplikationen: Organversagen, RR 0,27, 95%‐KI: 0,08–0,89). Für die Bewertung von Langzeitfolgen der Haut konnte eine Studie identifiziert werden, während für die Langzeitfolgen der Augen acht Studien für fünf direkte Paarvergleiche zur Verfügung standen (Tabelle S8 im Online‐Supplement).

Kein Vergleich erfüllte das OIS‐Kriterium.

## DISKUSSION

In der Literatur werden verschiedene systemische immunmodulierende Therapien für EN aufgeführt, aber keine davon zeigte einen eindeutigen Nutzen. In unsere Metaanalyse wurden 43 Studien mit 58 Therapievergleichen eingeschlossen. Neben der supportiven Therapie waren die am häufigsten eingesetzten Interventionen Kortikosteroide, IVIG, eine Kombination aus Kortikosteroiden und IVIG sowie Ciclosporin. Wir analysierten die Wirksamkeit der Interventionen in Bezug auf die Letalität, die Zeit bis zur vollständigen Reepithelisierung, die Dauer des Krankenhausaufenthalts, das Auftreten schwerwiegender Komplikationen und Langzeitfolgen.

Kortikosteroide sind zur Behandlung von EN weit verbreitet und stellen die häufigste SIT in Deutschland dar.^49^, ^64^ Bislang gibt es jedoch keine randomisierte kontrollierte Studie, die Kortikosteroide mit supportiver Therapie vergleicht. Vorhandene/vorliegende Kohortenstudien (meist retrospektive einarmige Studien und Fallberichte oder ‐serien) zeigten unterschiedliche Ergebnisse. Daher spiegeln unsere Ergebnisse die nach wie vor bestehende Debatte über den Einsatz von Kortikosteroiden wider. Frühere systematische Übersichtsarbeiten und Metaanalysen konnten keinen statistisch signifikanten Vorteil von Kortikosteroiden gegenüber supportiver Therapie in Bezug auf die Letalität nachweisen; lediglich Zimmermann et al. erzielten in einer Sekundäranalyse auf Patientenebene – unter Verwendung eines unstratifizierten Modells basierend auf individuellen Patientendaten – signifikante Ergebnisse (OR 0,67; 95%‐KI 0,46–0,97), während die Primäranalyse auf Studienebene keine statistisch signifikanten Resultate zeigte. (OR 0,54, 95%‐KI 0,29–1,01).^8^, ^9^, ^15^

In früheren Studien wurden häufig der Zeitpunkt des Therapiebeginns mit Kortikosteroiden, die Dosierung und die Behandlungsdauer sowie deren Auswirkungen auf Letalität, Infektionen und Dauer des Krankenhausaufenthalts thematisiert.^65^, ^66^, ^67^, ^68^ In dieser Metaanalyse haben wir uns gegen eine Differenzierung nach Dosis und Dauer der Kortikosteroidgabe entschieden, da die entsprechenden Daten in mehreren Studien unvollständig waren, was weiterführende Analysen stark eingeschränkt hätte.

Unsere Untersuchung von IVIG zur Behandlung von EN ergab keinen positiven Effekt im Vergleich zur supportiven Therapie in Bezug auf Letalität, Zeit bis zur vollständigen Reepithelisierung, Dauer des Krankenhausaufenthalts und schwerwiegender Komplikationen (Sepsis, Beatmung). Auch im Vergleich zu anderen SIT (insbesondere Ciclosporin und der Kombination von Kortikosteroiden plus IVIG) zeigten IVIG keinen Vorteil/Nutzen. Bereits frühere Metaanalysen konnten keine positiven Effekte, insbesondere hinsichtlich der Letalität, feststellen.^8^, ^9^, ^12^, ^16^

Unterschiedliche Dosierungen und Behandlungsprotokolle erschweren die Bewertung der IVIG‐Wirksamkeit – die Debatte darüber ist noch nicht abgeschlossen. Einige Studien fanden einen Vorteil hinsichtlich der Letalität bei Verwendung von hohen IVIG‐Gesamtdosen (≥ 2 g/kg KG),^69^, ^70^ während andere eine höhere Letalität als erwartet beobachteten.^71^ Eine Analyse niedriger versus hoher Dosen war in unserer Metaanalyse nicht möglich, da nur eine unzureichende Anzahl von Studien die Dosierung spezifizierte.

Entgegen der fehlenden Evidenz für die Wirksamkeit von Monotherapien gegenüber der supportiven Therapie zeigte die Kombination von Kortikosteroiden plus IVIG in unserer Analyse einen statistisch signifikanten Vorteil gegenüber IVIG allein bei der Letalität sowie gegenüber Kortikosteroiden bei der Zeit bis zur vollständigen Reepithelisierung. In früheren Metaanalysen konnte jedoch kein statistisch signifikanter Vorteil der Kombination von Kortikosteroiden plus IVIG nachgewiesen werden.^9^, ^12^, ^15^, ^72^ Wie bei den Monotherapien existieren auch für diese Kombinationstherapie keine Standardprotokolle, weshalb eine erhebliche Variabilität bei den Behandlungsmethoden vorliegt.

Verschiedene Metaanalysen berichten über eine niedrigere Letalität unter Ciclosporin im Vergleich zur supportiven Therapie, weshalb Ciclosporin als vielversprechende SIT gilt.^8^, ^9^, ^15^, ^18^, ^73^ In unserer Analyse wurde jedoch nur eine Studie in diesen Vergleich eingeschlossen, welche einen positiven Effekt auf die Zeit bis zur vollständigen Reepithelisierung zeigte.

Entgegen der fehlenden Evidenz für die Wirksamkeit von sowohl IVIG als auch Ciclosporin im Vergleich zur supportiven Therapie hinsichtlich der Letalität, ergab der direkte Vergleich von Ciclosporin mit IVIG einen statistisch signifikanten Vorteil zugunsten von Ciclosporin. Dieses Ergebnis ist jedoch mit Vorsicht zu interpretieren, da ein Nachweis der Überlegenheit von Ciclosporin gegenüber der alleinigen supportiven Therapie fehlt.

Aufgrund einer erhöhten Expression von TNF‐α in Proben betroffener Patienten^74^, ^75^, ^76^ kommen TNF‐α‐Inhibitoren zur Behandlung von EN zum Einsatz,^23^, ^24^, ^25^, ^34^, ^35^, ^46^, ^61^ obwohl die pathogenetischen Implikationen bislang nicht abschließend geklärt sind.^77^ Eine in unsere Metaanalyse einbezogene RCT mit Thalidomid, welche insgesamt 22 Patienten mit EN einschloss, wurde jedoch vorzeitig abgebrochen, da in der Thalidomid‐Gruppe eine erhöhte Letalität festgestellt wurde, die paradoxerweise mit erhöhten TNF‐α‐Spiegeln korrelierte.^23^ Basierend auf kleinen Fallzahlen unterhalb der OIS‐Schwelle zeigten die beiden anderen in unsere Analyse berücksichtigten TNF‐α‐Inhibitoren Etanercept und Adalimumab statistisch signifikante Behandlungseffekte.^24^, ^35^ Bereits vorliegende Metaanalysen berichten von positiven Effekten für TNF‐α‐Inhibitoren, diese basieren jedoch auf Fallberichten, Fallserien und/oder einer einzigen RCT mit Vergleich von Kortikosteroiden und Etanercept.^9^, ^12^, ^16^, ^78^

### Limitationen

Klinische Studiendaten zu EN sind rar. Die Qualität der Primärstudien ist im Allgemeinen mangelhaft im Hinblick auf methodische Genauigkeit und Berichtsstandards. Entscheidungen darüber, welche Studien in Metaanalysen berücksichtigt werden, beruhen daher nicht nur auf dem Wunsch, die Datenqualität nicht zu beeinträchtigen, sondern stellen einen Balanceakt zwischen Datenqualität und statistischer Aussagekraft dar. In nichtrandomisierten Interventionsstudien (NRSI) ist *Confounding* eine wesentliche Quelle für Verzerrungen (*Bias*) und erfordert eine sorgfältige Abwägung von Faktoren, die sowohl die Behandlungszuweisung als auch das gewünschte Ergebnis beeinflussen. Wir entschieden uns für die Berücksichtigung von NRSI, bezogen jedoch nur Studien mit einem minimalen Risiko für *Confounding* ein. Hierzu schlossen wir einarmige Studien aus, da diese nicht nur die Behandlungseffekte im Zusammenhang mit der betreffenden Intervention widerspiegeln, sondern auch zentrumsspezifische Effekte, wie etwa Unterschiede in der supportiven Therapie. Studien, bei denen klinisch bedeutsame Unterschiede in therapie‐ und ergebnisrelevanten Patientencharakteristika vorlagen, wurden ausgeschlossen. Da die Fähigkeit, diese Unterschiede zu identifizieren, von der Stichprobengröße und letztlich vom willkürlichen Schwellenwert für die klinische Relevanz abhängt, könnten dennoch Studien mit unentdeckten Störfaktoren (*Confoundern*) eingeschlossen worden sein. Andererseits könnten einarmige Studien, die detaillierte Informationen über potenzielle Störfaktoren (zum Beispiel SCORTEN‐Punktwert und supportive Behandlung) liefern, trotz ihrer möglichen Kombinierbarkeit mit kontrollierten NRSI‐Studien ausgeschlossen worden sein, woraus sich ein weiterer Verlust an statistischer Aussagekraft ergeben haben könnte.

### Schlussfolgerungen

Basierend auf unserer Metaanalyse publizierter Studien fehlen weiterhin Beweise für die Überlegenheit einer der routinemäßig eingesetzten systemischen immunmodulatorischen Behandlungen gegenüber der supportiven Therapie. Unsere Auswertung unterstreicht die Bedeutung qualitativ hochwertiger Forschungsdaten für die Behandlung seltener Krankheiten wie EN. Keine der untersuchten Behandlungsoptionen zeigte eine klare Überlegenheit gegenüber anderen, wenn man die Letalität, die Zeit bis zur Reepithelisierung oder die Dauer des Krankenhausaufenthalts betrachtet. Selbst wenn systemische Therapien letztlich wirksam sein sollten, ist es aufgrund der geringen Zahl an Studienteilnehmern, der mangelhaften Berichterstattung über Basischarakteristika und Behandlungsergebnisse, der heterogenen Studienpopulationen sowie der Begleittherapien nahezu unmöglich, kleine, aber tatsächlich existierende Behandlungseffekte in Metaanalysen von NRSI zu erkennen. Dies erfordert konzertierte Anstrengungen bei der Konzeption großer multizentrischer Studien, die höheren methodischen und Berichtsstandards entsprechen.

## DANKSAGUNG UND FINANZIELLE UNTERSTÜTZUNG

*Finanzierung/Unterstützung*: Diese Forschung wurde durch den Gemeinsamen Bundesausschuss (G‐BA) der gemeinsamen Selbstverwaltung der Ärzte, Zahnärzte, Krankenhäuser und Krankenkassen in Deutschland unter dem Förderkennzeichen 01VSF21002 gefördert.

*Rolle des Geldgebers/Sponsors*: Die Finanzierungsquelle hatte keinen Einfluss auf die Konzeption und Durchführung der Studie, die Erhebung, Verwaltung, Analyse und Interpretation der Daten, die Vorbereitung, Überprüfung oder Genehmigung des Manuskripts und die Entscheidung, das Manuskript zur Veröffentlichung einzureichen.

## DANKSAGUNG

Wir danken allen Autoren, die zusätzliche Informationen zu ihren Artikeln bereitgestellt haben.

Open access Veröffentlichung ermöglicht und organisiert durch Projekt DEAL.

## INTERESSENKONFLIKT

M. M. berichtete über ihre Tätigkeit als Mitglied eines wissenschaftlichen Beirats für Boehringer Ingelheim sowie als Beraterin für Pfizer und den Erhalt von Honoraren; den Erhalt von Zahlungen (an die Universität Freiburg) von mehreren Pharmaunternehmen (einschließlich Biogen, Janssen) für die Bereitstellung von Informationen zur Arzneimittelsicherheit sowie über den Erhalt von Zahlungen für ärztliche Fortbildungsarbeiten im Zusammenhang mit Nebenwirkungen von Arzneimitteln von der Dermatologischen Fortbildungs‐Gesellschaft mbH, Galderma und RG‐Fortbildungsveranstaltungen. R. H., M. P. und A. N. erklären, dass im Zusammenhang mit der vorliegenden Arbeit keine potenziellen Interessenkonflikte bestehen.

## Supporting information



Supplementary information
